# Harness machine learning for multiple prognoses prediction in sepsis patients: evidence from the MIMIC-IV database

**DOI:** 10.1186/s12911-025-02976-y

**Published:** 2025-03-31

**Authors:** Su-Zhen Zhang, Hai-Yi Ding, Yi-Ming Shen, Bing Shao, Yuan-Yuan Gu, Qiu-Hua Chen, Hai-Dong Zhang, Ying-Hao Pei, Hua Jiang

**Affiliations:** 1 Affiliated Hospital of Nanjing University of Chinese Medicine, Nanjing, Jiangsu Province China; 2 Department of Intensive Care Unit, Jiangsu Province Hospital of Chinese Medicine, Affiliated Hospital of Nanjing University of Chinese Medicine, 155 Han Zhong Road, Nanjing, Jiangsu Province China

**Keywords:** Sepsis, Chronic critical illness, Machine learning, Predictive model, Model interpretability

## Abstract

**Background:**

Sepsis, a severe systemic response to infection, frequently results in adverse outcomes, underscoring the urgency for prompt and accurate prognostic tools. Machine learning methods such as logistic regression, random forests, and CatBoost, have shown potential in early sepsis prediction. The study aimed to create and verify a machine learning model capable of early prognostic identification of patients with sepsis in intensive care units (ICUs).

**Methods:**

Patients adhering to inclusion and exclusion criteria from the MIMIC-IV v2.2 database were divided into a training set and a validation set in a 7:3 ratio. Initially, we employed difference analysis to assess the significance of each variable and subsequently screened relevant features with multinomial logistic regression analysis. Logistic regression, random forest, and CatBoost algorithms were used to construct machine learning models to predict rapid recovery, chronic critical illness, and mortality in sepsis. The models were compared through several evaluation indexes including precision, accuracy, recall, F1 score, and the area under the receiver-operating-characteristic curve(AUC) in the validation set to select the optimal model. The best model was visualized and interpreted utilizing the Shapley Additive explanations method.

**Results:**

13174 sepsis patients were included. Post the screening process,26 clinical features were obtained to develop three distinct machine learning models. CatBoost exhibited superior performance among the three models with a weighted AUC of 0.771. The prognosis with the highest predictive performance was mortality (AUC = 0.804), followed by the prognoses of rapid recovery (AUC = 0.773) and chronic critical illness(AUC = 0.737). Urine output, respiratory rate, and temperature were the top three important features for the whole model prediction.

**Conclusion:**

The machine learning model developed leveraging the CatBoost algorithm demonstrates the latent capacity to identify sepsis prognosis early. It also suggests that interventions targeting factors such as urine output, respiratory status, and temperature in the early stage may potentially alter the adverse prognosis of sepsis patients. However, the model will still require further external validation in the future.

**Supplementary Information:**

The online version contains supplementary material available at 10.1186/s12911-025-02976-y.

## Background

Sepsis, characterized as a detrimental systemic response to infection, poses a significant risk for the occurrence of life-threatening organ dysfunction [1]. With the condition advancing, it may escalate to multiple organ failure and ultimately fatality, especially if not identified swiftly and treated promptly. Sepsis is a leading cause of significant morbidity and mortality in intensive care units (ICUs), with a high incidence of disability among survivors. According to a research for the Global Burden of Disease, there were approximately 48.9 million cases of sepsis worldwide in 2017, resulting in 11 million deaths, which accounted for 19.7% of the global death toll that year [2]. An epidemiological survey in Chinese ICUs indicated that the incidence of sepsis was 20.6%, and the 90-day mortality rate was 35.5% [3]. With the improvement of life-saving techniques, a portion of the population survive the early acute phase and enter the chronic stage, known as Chronic Critical Illness (CCI) [4]. Research from Japan found that among 2395,016 patients admitted to ICU, 9.0% met the criteria for CCI, with sepsis being the underlying cause in 50.6% of those [5]. Patients with CCI often require long-term intensive care and stay in the ICU, leading to substantial consumption of healthcare resources [6, 7]. They frequently encounter challenges such as persistent inflammatory response [8], acquired immunosuppression [9], and hypercatabolism [10], which result in recurrent infections, prolonged hospitalization, and a markedly diminished quality of life.

Conventional prognostic prediction of sepsis is commonly assessed by clinical scoring systems such as Sequential Organ Failure Assessment (SOFA), quick SOFA (qSOFA), Systemic Inflammatory Response Syndrome (SIRS), and Acute Physiology and Chronic Health Evaluation II (APACHE II) [11–14]. Nevertheless, sepsis frequently involves multiple organ dysfunctions, contains lots of clinical information, and the diseased organs differ from person to person. So predicting the disease events using the traditional assessment methods may lead to results bias. The advent of machine learning(ML) algorithms in recent years has facilitated the prediction of disease events based on large and complex clinical information. Premised on the effective management of AI-associated risks by conforming to the European AI Act, machine learning holds the potential to render substantial contributions to disease prediction and treatment decision-making [15]. Advanced ML algorithms are adept at analyzing intricate signals in data-rich environments. Therefore, based on the advantages of analyzing big data, ML approaches are promising in sepsis prognostic prediction. Moreover, the integration of ML methods with epidemiology also represents an emerging trend, with the potential to be utilized across a broad spectrum of infectious disease research [16].

Currently, the prognosis of sepsis is commonly predicted based on logistic regression(LR) to construct nomogram [17–19]. These models are evaluated by comparing with classical scores such as SOFA and SAPS II, whose performance are often inferior to the models. However, this way frequently lacks comparison between models and has inherent limitations. Another approach involves constructing prognostic models through more flexible ML algorithms including ensemble learning, such as random forests(RF) [20, 21], support vector machines (SVM) [22, 23], extreme gradient lift (XGBoost) [24, 25], etc. And focus on the comparison of the performance of multiple models, such models can deal with more complex data structures, but sometimes not as explanatory as the former. Additionally, we found that most prognostic models were binary models, designed to predict whether sepsis patients die or develop into CCI. Since both of the two prognoses have a serious impact on the quality of life, we attempted to establish a model that can predict these two adverse outcomes concurrently to aid clinical decision-making.

This study was designed to construct a novel prediction model of multiple prognoses in sepsis based on ML methods using data from the MIMIC-IV v2.2 database and to facilitate early clinical intervention. We selected logistic regression(LR), random forest(RF), and CatBoost for training. LR was chosen for its widespread use, RF for its generally excellent performance, and CatBoost for its use of oblivious trees as base learners, which effectively reduces overfitting and offers high precision and robustness. CatBoost is particularly adept at multi-task learning and handling imbalanced datasets through its built-in balancing strategies.

## Methods

### Study design

**Fig. 1:**
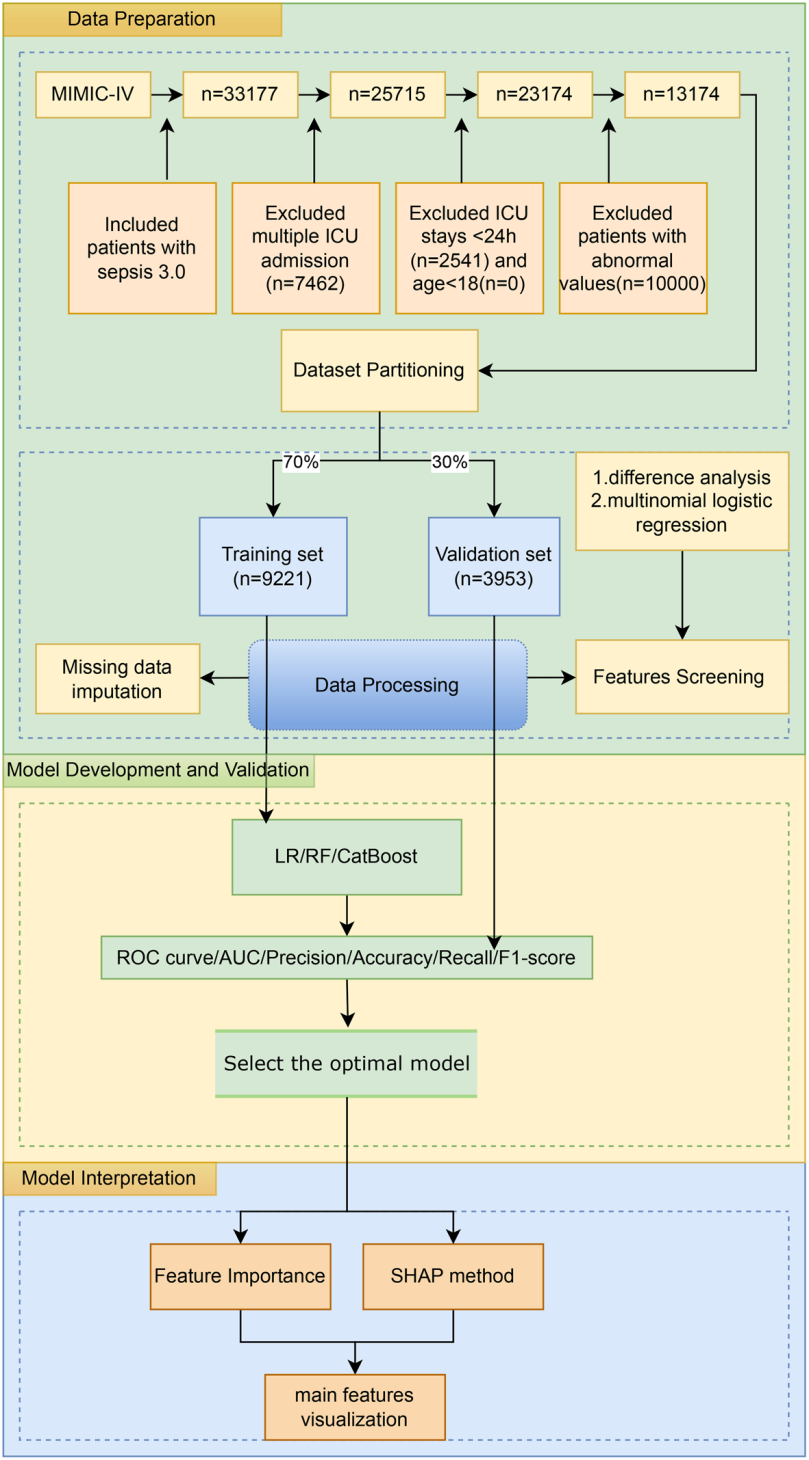
Schematic of the study workflow

This was a retrospective study and three ML algorithms were employed to train the models. Subsequent validation determined the most efficient algorithms. Additionally, the interpretability of the model was enhanced due to the use of the Shapley Additive explanations(SHAP) [26]. The detailed process of the study is shown in Fig. 1.

### Data sources and ethical review

Ethical concerns were thoroughly considered during the study design. The data utilized in this study were obtained from the MIMIC-IV v2.2, a database developed and maintained by MIT Laboratory of Computational Physiology. The database is the largest publicly accessible, privacy-free database in critical care medicine and contains comprehensive information on patients admitted to Beth Israel Deaconess Medical Center between 2008 and 2019. The database includes anonymized clinical information, ensuring that individual patient identities remain confidential [27]. Since the data come from a public database, it does not involve an ethical review. Three authors of this study have successfully completed the ethics training for the MIMIC database (one author with certification number: 12780309).

### Inclusion and exclusion criteria

Inclusion Criteria: ICU patients diagnosed with sepsis 3.0 for the first time:

i. suspected infection;
ii. SOFA ≥ 2.

Exclusion Criteria: i.Patients with multiple ICU admissions, for whom only initial admission data were considered; ii. Patients with an ICU stay shorter than 24 hours; iii. Patients under the age of 18 years; iv. Patients had no SOFA score within 24 hours after admission to the ICU;v.Patients had abnormal data or missed significant clinical information.

### Definition of CCI

For the definition of the CCI group, the diagnostic criteria of CCI were adopted from the Research Triangle Institute (RTI) [28], consisting of an ICU stay for at least 8 days with one of 5 eligible clinical conditions: prolonged acute mechanical ventilation (i.e. mechanical ventilation for at least 96 hours in a single episode); tracheotomy; sepsis and other severe infections; severe wounds; and multiple organ failure, ischemic stroke, intercerebral hemorrhage or traumatic brain injury.

### Data extraction

We used Navicat Premium16 to write structured query language (SQL) to extract data from the MIMIC - IV v2.2 database. A total of 56 common clinical variables were extracted, including patients’ demographic information such as age, gender, weight, height, ICU types, time in and out of ICU, and time of death; the Charlson Comorbidity Index was used to account for comorbidities, considering that the risk of comorbidities is not simply the sum of the risks caused by individual diseases; vital signs including temperature, respiratory rate, heart rate, systolic blood pressure, diastolic blood pressure, and mean arterial pressure, with the mean values used to represent the average level of patients within 24 hours; laboratory examinations, where for indicators such as hemoglobin, platelet count, bicarbonate, blood calcium, base excess, pH, blood oxygen partial pressure, partial pressure of carbon dioxide, oxygenation index, lymphocyte count, lymphocyte percentage, and albumin, the minimum values within 24 hours were retained, while for hematocrit, white blood cell count, C - reactive protein, neutrophil count, anion gap, creatinine, blood urea nitrogen, blood chloride, blood glucose, blood sodium, blood potassium, international normalized ratio, prothrombin time, partial thromboplastin time, lactic acid, red blood cell distribution width, D - Dimer, and fibrinogen, the maximum values within 24 hours were retained, as the value of each indicator corresponded to the level of the patient’s test time point and we selected the worst values within 24 hours; ICU monitoring including urine output and central venous pressure monitoring; and related treatments such as mechanical ventilation, renal replacement therapy, diuretics, milrinone, epinephrine, vasopressin, norepinephrine, phenylephrine, dopamine, and dobutamine within the first 24 hours of ICU admission.

### Data processing

The variables with missing values greater than 25% were removed, and 1% and 99% quantiles were used to remove outliers in continuous variables. Variables whose outliers were laborious to remove using the above method were replaced by the median. Categorical variables with a category percentage of less than 5% or containing ambiguous classifications were removed. The retained variables were subsequently utilized for further analysis. To avoid data contamination, the dataset was first randomly divided into training and validation sets according to a 7:3 ratio. After that the data were filled with method of spline using the interpolate function in Python for the training and validation sets respectively, a piecewise imputation approach that better aligns with the structure of the data. And the data was normalized by MinMaxScaler.

### Variable selection

Difference analysis was applied to check the significance of each variable and multinomial logistic regression analysis was used to screen relevant features.

### Statistical analysis

Data analyses were conducted by Python software(version 3.7) and SPSS software (version 26.0). Continuous variables adhering to a normal distribution were described as the mean (standard deviation), and variables deviating from a normal distribution were described by median (interquartile range, IQR), none of the variables in this study conformed to normal distribution. Categorical variables were described by frequency (percentage). For between-group comparisons, the Kruskal-Wallis test was used for continuous variables; the chi-square test was used for categorical variables. Subsequently, multinomial logistic regression analysis was employed to screen features, The area under the receiver-operating-characteristic curve (AUC) was mainly used to evaluate the performance of the models. The threshold for statistical significance was set at *P* < 0.05.

### Machine learning methods

In this study, LR, RF, and CatBoost algorithms were used to develop prediction models. Open-source scikit-learn(http://scikitlearn.org/) was used for model construction, tuning, validation, and results interpretation in Python software(version 3.7).

The data was divided into a training set and validation set with a ratio of 7:3 by stratified method, and the models were constructed by the training set and validated by the validation set. Taking 7 days as the time node, discharge within 7 days, death within 7 days, and development into CCI were taken as the three prognoses of sepsis: i.e., rapid recovery, mortality, and CCI. And use the three prognoses as the ending index to construct the classification models. To optimize the prediction models, randomized search combined with manual fine-tuning was applied to obtain the final hyperparameters. Considering the issue of imbalance in the dataset, we chose to use the model’s built-in weight parameter to balance the distribution (i.e., setting class_weight = “balanced”). When set to “balanced”, the model automatically adjusts the weights of the groups, making the weight of each group inversely proportional to its sample size. This gives higher weights to samples of the minority group during training, thereby balancing the impact of each class. The core of this approach lies in adjusting the loss function so that the optimization process takes into account all groups in a more balanced manner. Precision, Accuracy, Recall, F1-score, and the area under the ROC curve (AUC) were calculated to evaluate the models. Some studies have demonstrated that interpretability and transparency remain challenges for ML [29]. We introduced SHAP method to enhance the interpretability of the model, which can effectively reveal the intrinsic logic behind model predictions.

## Results

### Participants

A total of 33177 records of patients admitted to ICU who met the diagnostic criteria for sepsis 3.0 were obtained from the MIMIC-IV v2.2 database. 25,715 patients were obtained by removing records of repeated admission. 23,174 patients were included for ICU stays of more than 24 hours, and 13174 patients were finally obtained by removing records of patients with abnormal values (Fig. 1).

### Feature screening result

Following data processing, 37 out of the 56 variables were retained. Table 1 shows 37 variables with significant differences in distribution among the three groups through difference analysis.

**Table 1:** Difference analysis of variables in three groups of rapid recovery, CCI, and mortality

	Rapid Recovery group	CCI group	Mortality group	Overall	*P*-value
	*n* = 9170	*n* = 2843	*n* = 1161	*n* = 13174	
Gender					
Female	3556(38.78%)	1200(42.21%)	541(46.60%)	5297(40.21%)	<0.001
Male	5614(61.22%)	1643(57.79%)	620(53.40%)	7877(59.79%)	
Mechanical ventilation					
Yes	4563(49.76%)	1877(66.02%)	625(53.83%)	7065 (53.63%)	<0.001
No	4607(50.24%)	966(33.98%)	536(46.17%)	6109 (46.37%)	
CVP monitoring					
Yes	4341(47.34%)	949(33.38%)	352(30.32%)	5642 (42.83%)	<0.001
No	4829(52.66%)	1894(66.62%)	809(69.68%)	7532 (57.17%)	
Norepinephrine					
Yes	1992(21.72%)	1129(39.71%)	584(50.30%)	3705 (28.12%)	<0.001
No	7178(78.28%)	1714(60.29%)	577(49.70%)	9469 (71.88%)	
Phenylephrine					
Yes	3215(35.06%)	777(27.33%)	312(26.87%)	4304 (32.67%)	<0.001
No	5955(64.94%)	2066(72.67%)	849(73.13%)	8870 (67.33%)	
Epinephrine					
Yes	482(5.26%)	176(6.19%)	79(6.80%)	737 (5.59%)	0.028
No	8688(94.74%)	2667(93.81%)	1082(93.20%)	12437 (94.41%)	
Age	67.64(57.09,77.58)	65.86(53.82,76.13)	72.91(61.75,82.48)	67.64(56.79,77.77)	<0.001
Charlson Comorbidity Index	5.00(4.00,7.00)	6.00(4.00,8.00)	7.00(5.00,9.00)	6.00(4.00,8.00)	<0.001
Weight, kg	80.00(68.00,95.00)	81.40(68.00,97.95)	76.20(63.00,90.00)	80.00(67.50,95.00)	<0.001
Urine output, mL	1723.00(1115.00,2480.00)	1415.00(804.50,2242.50)	910.00(375.00,1635.00)	1590.00(970.00,2385.00)	<0.001
HR_mean,bpm	83.94(75.93,94.60)	89.00(77.34,101.96)	92.08(79.11,104.72)	85.34(76.36,97.33)	<0.001
RR_mean,bpm	18.25(16.30,20.84)	20.06(17.60,23.38)	21.18(18.16,24.36)	18.81(16.63,21.77)	<0.001
SBP_mean, mm Hg	113.04(105.86,121.48)	113.13(104.54,125.21)	108.39(100.91,118.63)	112.63(105.12,121.97)	<0.001
DBP_mean, mm Hg	59.52(54.34,65.38)	60.72(54.84,67.67)	59.17(52.77,66.56)	59.73(54.34,66.00)	<0.001
MAP_mean, mm Hg	75.10(70.58,80.96)	76.07(70.62,83.47)	73.40(67.43,80.58)	75.19(70.32,81.44)	<0.001
Temp_mean,℃	36.85(36.59,37.16)	37.05(36.66,37.46)	36.78(36.45,37.22)	36.87(36.59,37.24)	<0.001
HCT_max, %	34.00(30.40,38.10)	35.30(30.70,40.10)	34.20(29.40,39.20)	34.30(30.40,38.60)	<0.001
WBC_max, × 103/μL	14.40(10.70,19.00)	14.80(10.70,19.80)	15.50(10.60,21.20)	14.50(10.70,19.30)	<0.001
Hb_min,g/dL	9.50(8.20,10.90)	9.80(8.30,11.60)	9.40(7.90,11.10)	9.60(8.20,11.00)	<0.001
PLT_min, × 103/μL	150.00(109.00,209.00)	162.00(107.00,223.00)	150.00(84.00,224.00)	153.00(107.00,214.00)	<0.001
Chloride_max, mmol/L	108.00(104.00,111.00)	107.00(103.00,111.00)	106.00(101.00,111.00)	108.00(104.00,111.00)	<0.001
Sodium_max, mmol/L	140.00(138.00,142.00)	141.00(138.00,143.00)	140.00(137.00,144.00)	140.00(137.00,143.00)	<0.001
Potassium_max, mmol/L	4.50(4.20,4.90)	4.50(4.10,5.10)	4.70(4.20,5.30)	4.50(4.10,5.00)	<0.001
Calcium_min, mmol/L	8.00(7.50,8.40)	7.80(7.30,8.30)	7.80(7.20,8.40)	7.90(7.40,8.40)	<0.001
Glucose_max, mg/dL	140.00(116.00,182.00)	163.00(131.00,212.00)	167.00(129.00,229.00)	146.00(119.00,193.00)	<0.001
BUN_max, mg/dL	20.00(14.00,32.00)	25.00(16.00,41.00)	36.00(22.00,57.00)	22.00(15.00,36.00)	<0.001
Cr_max, mg/dL	1.00(0.80,1.50)	1.20(0.80,2.00)	1.60(1.10,2.60)	1.10(0.80,1.70)	<0.001
INR_max	1.30(1.20,1.60)	1.40(1.20,1.70)	1.60(1.30,2.30)	1.40(1.20,1.60)	<0.001
PT_max,s	14.90(13.30,17.10)	14.90(12.90,18.70)	17.00(13.90,24.20)	15.00(13.30,17.80)	<0.001
PTT_max,s	32.20(28.50,39.48)	32.40(28.30,43.60)	35.40(28.50,49.60)	32.40(28.50,40.90)	<0.001
AG_max	15.00(12.00,18.00)	17.00(14.00,20.00)	19.00(16.00,23.00)	16.00(13.00,19.00)	<0.001
Lac_max,mmol/L	2.20(1.50,3.20)	2.30(1.50,3.90)	3.00(1.80,5.40)	2.20(1.50,3.40)	<0.001
Bicarbonate_min,mmol/L	22.00(19.00,24.00)	20.00(17.00,23.00)	19.00(15.00,22.00)	21.00(18.00,24.00)	<0.001
BE_min,mmol/L	−3.00(−5.00,0.00)	−4.00(−8.00,0.00)	−5.00(−10.00,-1.00)	−3.00(−6.00,0.00)	<0.001
pH_min	7.32(7.27,7.37)	7.30(7.22,7.37)	7.29(7.19,7.37)	7.32(7.26,7.37)	<0.001
PO_2__min, mm Hg	80.00(47.00,112.00)	67.00(44.00,92.00)	52.00(37.00,80.00)	74.00(45.00,105.00)	<0.001
PCO_2__max, mm Hg	46.00(41.00,52.00)	47.00(41.00,56.00)	46.00(39.00,56.00)	46.00(41.00,53.00)	<0.001

The cohort was divided into 3 groups: rapid recovery, CCI, and mortality groups. The study utilized medians and quartiles to compare continuous variables and frequencies and percentages to compare component variables for statistical descriptions. The Kruskal-Wallis test was used for continuous variables; the chi-square test was used for categorical variables. HR_mean, heart rate mean, RR_mean, respiratory rate mean, SBP_mean, systolic blood pressure mean, DBP_mean, diastolic blood pressure mean, MAP_mean, mean arterial pressure mean, Temp_mean, temperature mean, HCT_max, hematocrit maximum, WBC_max, white blood cell count maximum, Hb_min, hemoglobin minimum, PLT_min, platelet count minimum, BUN_max, blood urea nitrogen maximum, Cr_max, creatinine maximum, INR_max, international normalized ratio maximum, PT_max, prothrombin time maximum, PTT_max, partial thromboplastin time maximum, AG_max, anion gap maximum, Lac_max, lactic acid maximum, BE_min, base excess minimum, PO2_min, blood oxygen partial pressure minimum, PCO2_max, partial pressure of carbon dioxide

After collinearity diagnosis of the above variables, the VIF of PT, INR, pH, and BE were greater than 10. Considering the close correlation between PT and INR, pH, and BE, one of them was removed respectively and INR and pH were retained. After collinearity diagnosis again, there was no obvious collinearity between the remaining variables. The remaining variables were analyzed using multinomial logistic regression analysis and 26 of them were selected (Table 2).

**Table 2:** Multinomial logistic regression analysis results

		CCI group				Mortality group			
		B	P	OR	95% CI	B	P	OR	95% CI
Intercept		−6.927	0.095			−9.74	0.087		
Age		−0.015	<0.001	0.985	0.981–0.989	0	0.942	1.000	0.994–1.006
Charlson Comorbidity Index		0.046	<0.001	1.047	1.025–1.069	0.120	<0.001	1.128	1.097–1.160
Weight, kg		0	0.891	1.000	0.998–1.002	−0.010	<0.001	0.990	0.986–0.994
Urine output, mL		−0.023	<0.001	0.977	0.973–0.982	−0.035	<0.001	0.966	0.959–0.973
HR_mean,bpm		0.005	0.007	1.005	1.001–1.008	0.011	<0.001	1.011	1.006–1.016
RR_mean,bpm		0.081	<0.001	1.084	1.071–1.098	0.106	<0.001	1.112	1.093–1.132
SBP_mean, mm Hg		0.001	0.744	1.001	0.996–1.006	−0.007	0.107	0.993	0.985–1.001
DBP_mean, mm Hg		−0.037	<0.001	0.964	0.953–0.975	−0.005	0.590	0.995	0.978–1.013
MAP_mean, mm Hg		0.046	<0.001	1.047	1.032–1.061	0.011	0.310	1.011	0.990–1.033
Temp_mean,℃		0.245	<0.001	1.278	1.171–1.395	−0.164	0.010	0.849	0.749–0.961
HCT_max, %		0.005	0.405	1.005	0.993–1.018	−0.022	0.021	0.978	0.959–0.997
WBC_max, × 103/μL		−0.013	<0.001	0.987	0.981–0.994	−0.002	0.658	0.998	0.989–1.007
Hb_min,g/dL		0.066	0.001	1.069	1.029–1.110	0.145	<0.001	1.156	1.093–1.223
PLT_min, × 103/μL		0	0.095	1.000	1.000–1.001	0	0.772	1.000	0.999–1.001
Chloride_max, mmol/L		−0.048	<0.001	0.953	0.936–0.970	−0.033	0.009	0.968	0.944–0.992
Sodium_max, mmol/L		0.059	<0.001	1.061	1.042–1.081	0.046	<0.001	1.047	1.021–1.074
Potassium_max, mmol/L		−0.067	0.054	0.935	0.873–1.001	−0.004	0.933	0.996	0.908–1.093
Calcium_min, mmol/L		−0.169	<0.001	0.845	0.795–0.898	−0.120	0.006	0.887	0.815–0.966
Glucose_max, mg/dL		0	0.149	1.000	1.000–1.001	0	0.894	1.000	0.999–1.001
BUN_max, mg/dL		0.011	<0.001	1.011	1.008–1.014	0.017	<0.001	1.017	1.013–1.021
Cr_max, mg/dL		−0.136	<0.001	0.873	0.832–0.917	−0.129	<0.001	0.879	0.824–0.938
INR_max		0.093	0.007	1.097	1.026–1.174	0.260	<0.001	1.297	1.202–1.399
PTT_max,s		0.003	0.003	1.003	1.001–1.006	0.006	<0.001	1.006	1.003–1.009
AG_max		0.003	0.757	1.003	0.985–1.021	0.007	0.581	1.007	0.982–1.033
Lac_max,mmol/L		0.022	0.143	1.023	0.992–1.054	0.155	<0.001	1.167	1.123–1.214
Bicarbonate_min,mmol/L		−0.024	0.038	0.976	0.954–0.999	−0.03	0.066	0.970	0.940–1.002
pH_min		−1.183	0.024	0.306	0.110–0.853	0.889	0.217	2.432	0.593–9.978
PO_2__min, mm Hg		0.001	0.357	1.001	0.999–1.002	0	0.963	1.000	0.998–1.002
PCO_2__max, mm Hg		0.005	0.240	1.005	0.997–1.012	0.020	<0.001	1.020	1.010–1.031
Gender	Female	0.065	0.196	1.068	0.967–1.179	0.129	0.082	1.138	0.984–1.316
	Male	0^b^	.	.	.	0^b^	.	.	.
Mechanical ventilation	Yes	0.727	<0.001	2.069	1.871–2.288	0.388	<0.001	1.473	1.267–1.714
	No	0^b^	.	.	.	0^b^	.	.	.
CVP monitoring	Yes	−0.510	<0.001	0.600	0.537–0.672	−0.697	<0.001	0.498	0.422–0.588
	No	0^b^	.	.	.	0^b^	.	.	.
Norepinephrine	Yes	0.584	<0.001	1.793	1.599–2.011	0.679	<0.001	1.972	1.675–2.320
	No	0^b^	.	.	.	0^b^	.	.	.
Phenylephrine	Yes	0.067	0.257	1.070	0.952–1.202	0.174	0.049	1.190	1.001–1.415
	No	0^b^	.	.	.	0^b^	.	.	.
Epinephrine	Yes	0.206	0.056	1.229	0.995–1.518	0.024	0.876	1.025	0.755–1.390
	No	0^b^	.	.	.	0^b^	.	.	.

Note: The rapid recovery group was used as a reference, and the CCI group and Mortality group were compared with the rapid recovery group.0^b^ indicates reference category in categorical variables. Cox & Snell R-square = 0.217 Likelihood ratio test *p* < 0.001. Bolded variables are meaningful variables

### Model development and validation

Samples were divided into training and validation sets with a ratio of 7:3 through the stratified method (Table 3). Subsequently, three models were developed by the training set and assessed using the validation set. The values of evaluation indexes for 3 algorithms are illustrated in Table 4.

**Table 3:** Data set dividing information

Prognoses	Training set	Validation set	Overall
Rapid recovery	6418	2752	9170
CCI	1990	853	2843
Mortality	813	348	1161
Overall	9221	3953	13174

**Table 4:** Performance of the three models in the validation set

Models	Precision (95%CI)	Accuracy (95%CI)	Recall (95%CI)	F1-score (95%CI)	AUC (95%CI)
LR	0.672(0.655, 0.689)	0.619(0.604, 0.634)	0.619(0.603, 0.634)	0.636(0.621, 0.652)	0.747(0.732, 0.760)
RF	0.666(0.647, 0.686)	0.714(0.700, 0.728)	0.714(0.700, 0.730)	0.664(0.647, 0.682)	0.755(0.742, 0.769)
CatBoost	0.684(0.669, 0.701)	0.652(0.637, 0.666)	0.652(0.637, 0.666)	0.665(0.650, 0.679)	0.771(0.758, 0.785)

Note: Due to the unbalanced classification of this dataset, we adopted a weighted calculation method for the above multiple classification indexes and assigned different weights to each group

**Fig. 2:**
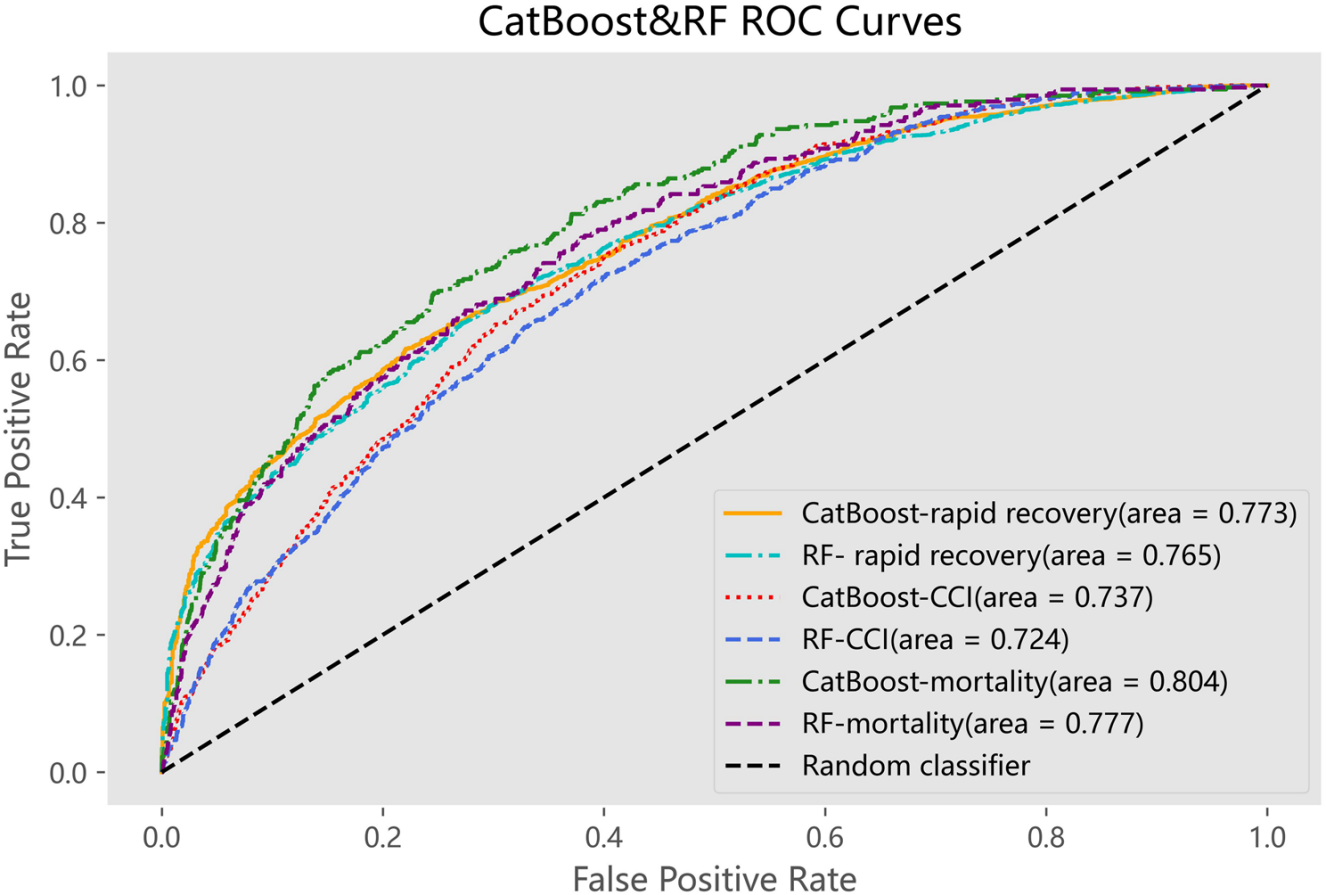
ROC curves for rapid recovery, CCI and mortality of Catboost and RF

**Fig. 3:**
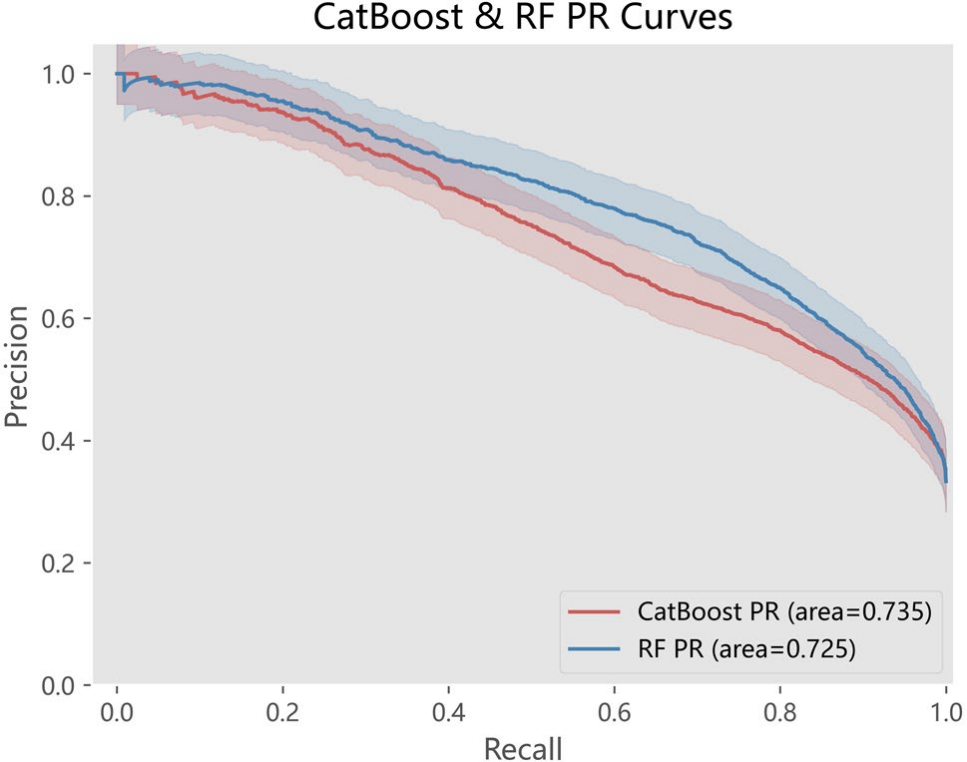
Precision-Recall curves of Catboost and RF

The models exhibiting superior performance were CatBoost and random forest, with respective weighted AUC in the validation set of 0.771 and 0.755, both higher than 0.747 of the logistic regression (Table 4). To further evaluate the performance of the two superior models for each prognosis, we generated ROC curves for each prognosis respectively (Fig. 2). Both of them predicted the prognosis of mortality better than the other two, in line with the prioritization of clinical decision-making. The difference in prediction performance between RF and CatBoost was insignificant, but the CatBoost model was better, where CatBoost reached an AUC of 0.804 for mortality prediction. This result was similar to the predictive performance observed in other studies on mortality rates for infectious diseases [30]. Moreover, the precision-recall(P–R) curves in Fig. 3 for Random Forest and CatBoost showed minimal difference, yet CatBoost demonstrates a slight advantage due to its superior handling of imbalanced datasets. To better evaluate the performance of the model, we further plotted the ROC and PR curves for the clinical scores commonly used in the Additional file 1 and compared them with CatBoost (Supplementary Figure 2). It can be seen that these clinical scores have a certain predictive value for adverse outcomes, with APACHE II being particularly prominent. When compared with CatBoost, the latter showed relatively better predictive performance for mortality prognosis and also had a good predictive effect on the rapid recovery group.

### Interpretation of the optimal model

**Fig. 4:**
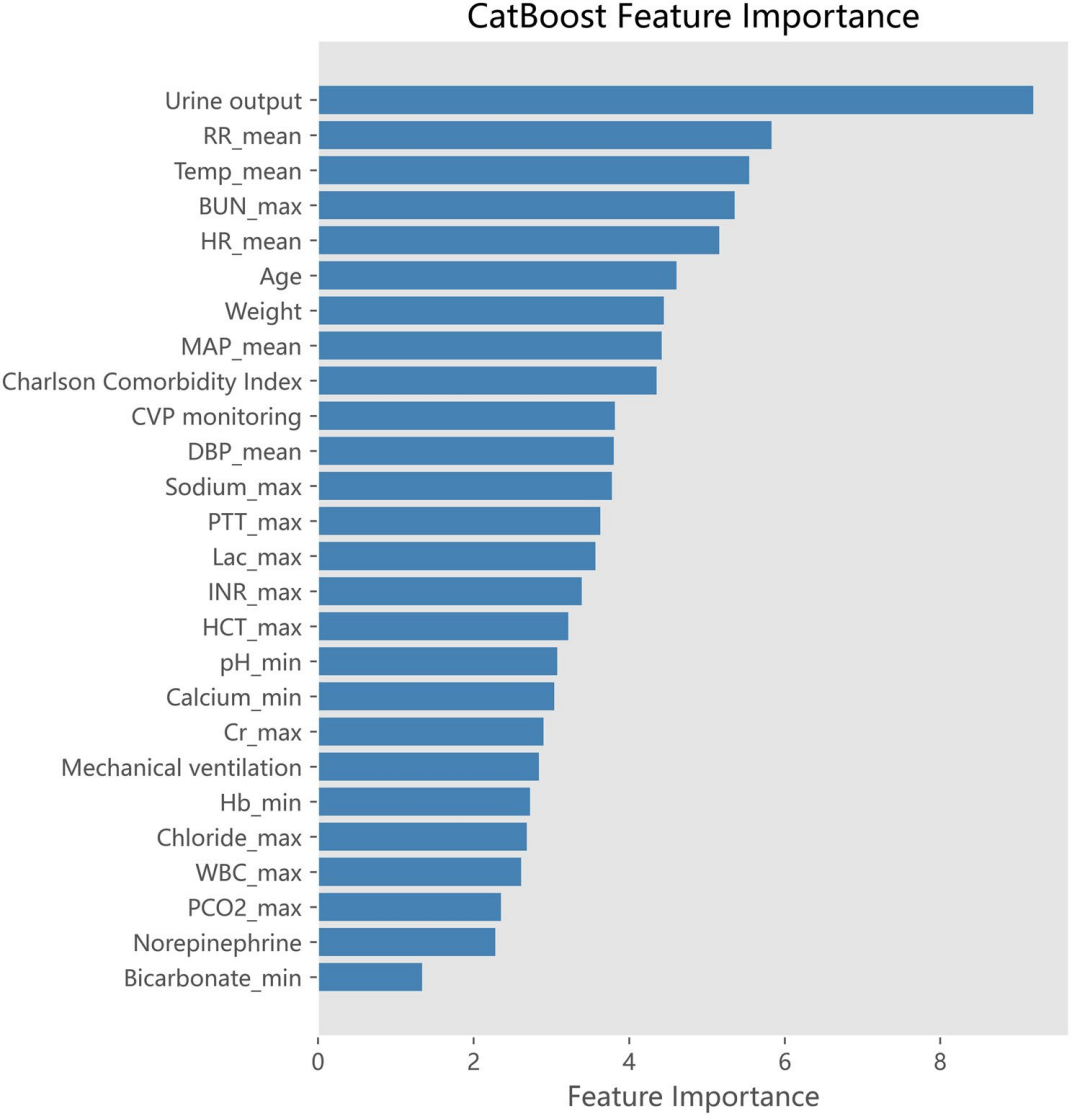
Ranking of CatBoost feature importance

**Fig. 5:**
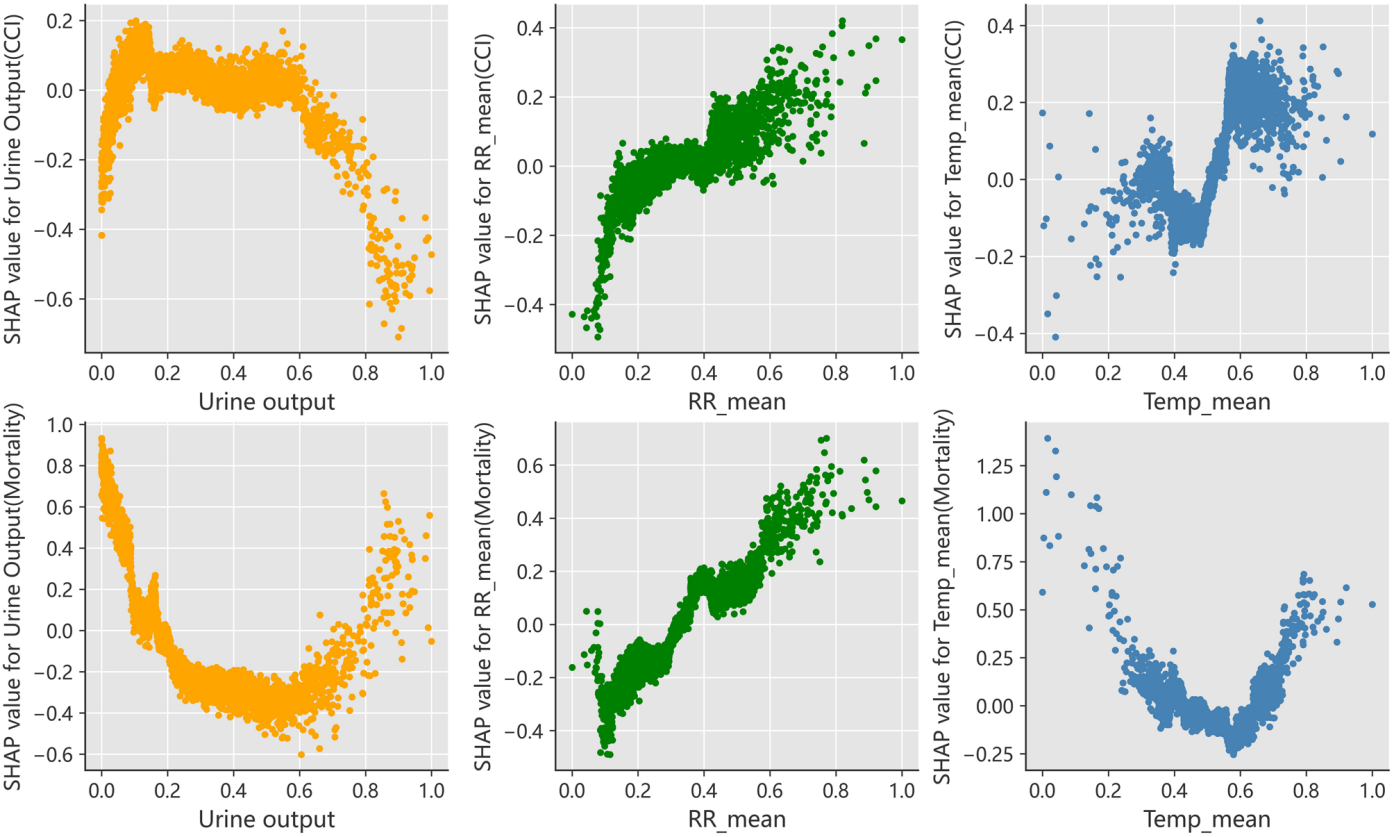
Shap dependence plots of urine output, respiratory rate, and temperature. Show the marginal effects of the three in the prognoses of CCI and mortality(Data normalized). Respiratory rate shows an almost monotonic change in both outcomes, while urine output and body temperature exhibit complex “U-shaped” variations

The best model was CatBoost. Through ranking of feature importance derived from CatBoost, our investigation revealed that three clinically-relevant feature - urine output, respiratory rate, and temperature - emerged as the most influential predictors of sepsis prognosis (Fig. 4). Furthermore, to enhance the interpretability of nonparametric models, which often lack transparency, we employed SHAP method for a visual representation of the features importance. This approach enables us to quantify each feature’s contribution to both the overall and specific prediction of the model. The SHAP dependence plots were drawn to examine how the three main features contribute to model prediction. The influence tendency of them on the model was complex (Fig. 5).

**Fig. 6:**
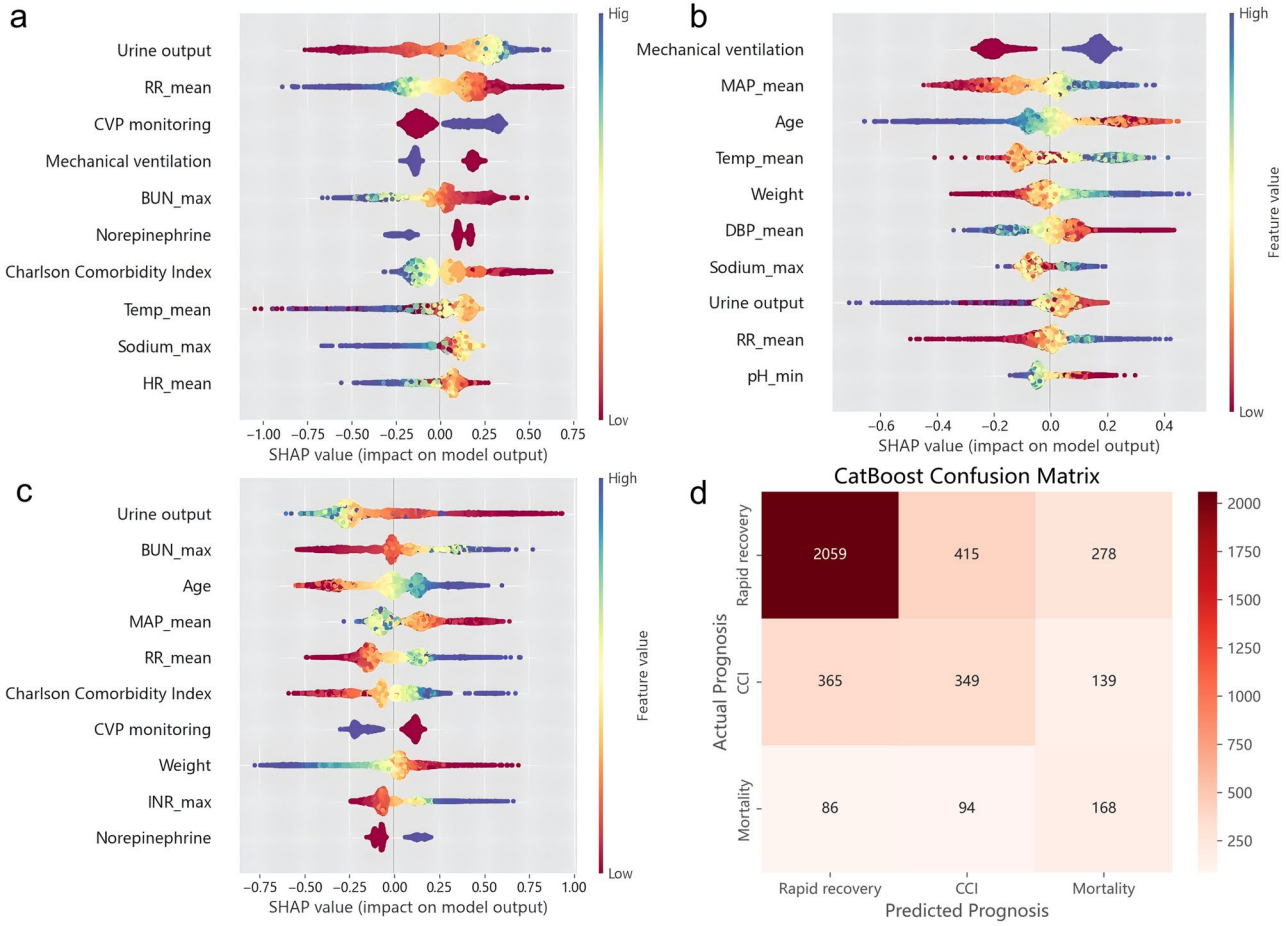
Beeswarm plots of different prognoses and confusion matrix predicted by CatBoost. **a** Beeswarm plot of prediction of rapid recovery. **b** Beeswarm plot of prediction of CCI. **c** Beeswarm plot of prediction of mortality. **d** Confusion matrix of CatBoost

**Fig. 7:**
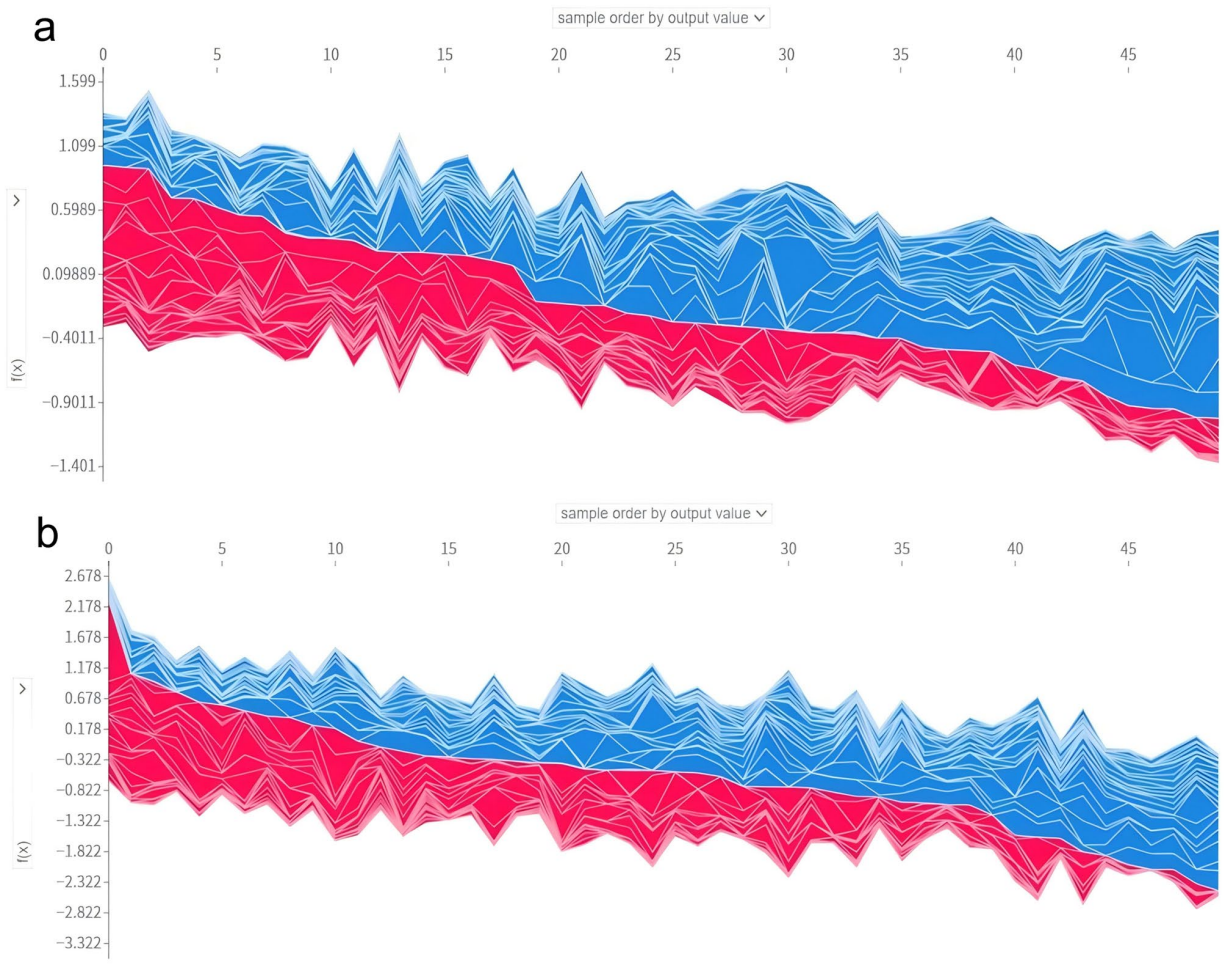
Force plots for the validation set. **a** Force plot for the cohort of CCI. **b** Force plot for the cohort of mortality. Each patient was represented by the x-axis, while the features contributions were represented by the y-axis: an increased red part for each patient represented a greater probability towards the prognosis of CCI or mortality

As shown in Fig. 6, it was evident that the main features in predicting various prognoses were distinct. With respect to CCI, the top three features were mechanical ventilation, MAP, and age, and for mortality were urine output, BUN, and age. In addition, the confusion matrix of the CatBoost model in the validation set is presented in Fig. 6d. Previously we obtained that CatBoost had the highest AUC for mortality prediction, but we also found that there was the possibility of overconfidence for the prediction of poor prognoses in the meantime(Rapid recovery was predicted to CCI and mortality in many cases) (Fig. 6d). Overconfidence in mortality prediction may lead to excessive clinical attention to these patients, potentially resulting in the irrational allocation of medical resources based on the severity of their conditions. However, it may also enhance the clinical vigilance of healthcare providers, potentially reducing mortality rates to some extent. And we performed model calibration using isotonic regression and present the results in Additional file 1 (Supplementary Table 1 and Figures 3 and 4). Initial calibration of the overall model also revealed that the predicted probabilities of mortality were higher than the actual, placing the calibration curves below the 45-degree line (Supplementary Figure 3). The metrics for the CatBoost model before and after calibration are detailed in Supplementary Table 1. The overall calibration did not improve the model’s mortality prediction. Following this, a mortality-specific recalibration was conducted, yielding a slight, albeit non-significant enhancement in calibration performance (Supplementary Figure 4). The model’s predictive capacity for rapid recovery outcome is constrained, likely due to the large population and complex individual differences within this patient cohort, which limits the predictive performance across different datasets. Moreover, during model training, the emphasis on the most adverse prognosis may indirectly contribute to the result. Furthermore, force plots of interpretation for 50 patients in CCI and mortality groups in the validation set are illustrated in Fig. 7. They show the combined contribution of each feature to prediction.

## Discussion

To date, sepsis remains a serious problem that jeopardizes human life and health. The combination of public databases and ML methods offers opportunities for research on sepsis diagnosis [31], complications and prognosis prediction [32–34], and treatment strategies [35, 36]. Undoubtedly, ensuring data security, patient privacy and adherence to AI ethics is of paramount importance whether using public databases or in the clinic. In this study, we endeavored to construct a prognostic prediction model of sepsis. Our findings indicated that the CatBoost model outperformed commonly employed models for sepsis prognosis prediction, achieving an AUC of 0.771 and an F1-score of 0.665.

We found that urine output, respiratory rate, and temperature played key roles in model forecasting. Urine output serves as an indicator of adequate perfusion. Sepsis-associated decreased urine output results from reduced renal perfusion due to systemic inflammation, capillary leak, and compensatory vasoconstriction. Pro-inflammatory cytokines and renal vasoconstriction further impair glomerular filtration and tubular function, while microvascular thrombosis exacerbates renal ischemia. This clinical manifestation is indicative of disease progression and worsening severity in sepsis. And beeswarm plots illustrate that individuals with higher urine output are more likely to recover, whereas those with lower output face a greater risk of mortality. It is similar to the findings of a study by Heffernan AJ et al [37] that low urine output means a high likelihood of death in sepsis patients. Through a meticulous examination of the 3 pivotal predictors (Fig. 5), we observed that urine output exhibited an opposite U-shaped association with CCI and mortality risk, suggesting a complex relationship with poor prognoses. Within a certain range, increased urine output was related to an increased risk of CCI and a decreased risk of mortality. However, scattered data points imply that excessively high urine output can also elevate the risk of mortality. Sepsis is associated with a reduction in circulating blood volume, which subsequently leads to the accumulation of acidic metabolites and triggers a compensatory increase in respiratory rate. Consequently, an elevated respiratory rate frequently signals the onset of clinical deterioration. Beeswarm plots similarly indicate that patients with fast respiratory rates are more likely to develop CCI or mortality (Fig. 6). The plots also reveal a monotonically positive correlation between respiratory rate and the risk of CCI and mortality (Fig. 5). The impact of body temperature on CCI and mortality was different, patients with very low body temperature were susceptible to die. Hypothermia in sepsis is caused by hypothalamic dysfunction and peripheral vasodilation due to infection. The former disrupts thermoregulation, while the latter increases heat loss. Metabolic depression resulting from impaired cellular metabolism and infectious effects further reduces heat production. These factors collectively lead to a significant drop in temperature. Hypothermia may impair immune function, exacerbate organ dysfunction, and disrupt inflammatory regulation, correlating with disease severity and adverse outcomes. A multicenter, large-sample study conducted by Saxena et al. also confirmed that sepsis patients with the lowest mortality risk were those who experienced high body temperatures like peak temperatures of 38–39.4 °C during the first 24 hours after ICU admission [38]. This suggests that a certain degree of initial hyperthermia may have a positive impact on patient prognosis. As body temperature rose, the risk of mortality initially decreased and subsequently increased, demonstrating that both excessively low and high body temperatures signify ominous prognosis (Fig. 5). Furthermore, those with very high body temperature were inclined to develop CCI as well. The reason may be that low temperature means a weakened immune system, predisposing patients to death, while high temperature is also detrimental due to ongoing inflammatory response and organ failures.

SHAP values offer an intuitive interpretation of model decisions. Although the variables highlighted by SHAP values as having significant predictive value do not have a direct causal relationship with the outcome, SHAP analysis indicates that variables such as urine output and respiratory status make substantial contributions to outcome prediction. Therefore, these variables warrant particular attention in the early stage. As evidenced by the results, patients exhibiting higher urine output, a relatively slow respiratory rate, and stable CVP monitoring are more likely to recover. For the three important predictors, the focus of CCI is principally on respiratory and circulatory status. Decreased urine output and elevated blood urea nitrogen are certainly of the essence to death outcome, underscoring the critical role of renal function in survival prognosis. This suggests that we should be extra vigilant for patients with poor renal function and closely monitor the level of urine output and renal function. Taken together, age has an obvious impact on adverse prognoses. Relatively younger patients are prone to develop CCI, while older patients tend to have a higher risk of death (Fig. 6). Meanwhile, the above features also reflect the importance of 24 - hour ICU monitoring and various monitoring methods such as in - out and CVP monitoring.

However, this study also has limitations. Firstly, due to the constraints of the database, the singularity of the dataset, regional epidemiology and so on bring certain limitations on the model’s generalizability. And some data in the database is incomplete and limits the inclusion indicators, potentially resulting in the loss of critical features and suboptimal model performance. Besides, for patients excluded due to the length of ICU stay less than 24 hours, these individuals may have been transferred, discharged, or unfortunately succumbed to their severe condition. Given that data for these patients within the first 24 hours were incomplete, we excluded them from the study. This exclusion may somewhat compromise the model’s predictive performance for this patient group. Additionally, the distribution of data sets was imbalanced. Although weights were applied to balance the distribution of different prognoses in data sets, the model’s predictive performance might still be affected. Moreover, this study was retrospective, and the model was internally verified using data set partitioning but not externally verified. Furthermore, the absence of definitive evidence of the onset of sepsis means that the levels of single cross-sectional biomarkers obtained at the earliest time point considered to be associated with clinical manifestation also brings limitations, as the timing of initial clinical presentation may affect the dynamics of the biomarkers [39]. The probable reason for this is that the appearance of clinical manifestations may not coincide with the initiation of sepsis.

This study offers insights into the development of a multi-classification prediction model for sepsis. However, when any model is used in clinical decision-making, external validation, prospective validation, and randomized clinical trials are essential to make rational judgments [40]. The lack of external validation somewhat restricts our evaluation of the model’s generalization ability. In the future, we will be committed to conducting multi-center clinical study and simultaneously collecting available retrospective clinical data to obtain sufficient external validation data, thereby thoroughly evaluating the generalizability and clinical performance of the model. If the model is verified in clinical practice, it will be convenient to build a website or develop a simple predictive tool for its application in ICU subsequently. Additionally, model explainability is crucial for understanding, trusting, and applying the model. In our paper, we employed methods such as the confusion matrix and SHAP plots to enhance the explainability of the model. Despite these efforts, tools for interpreting ML models remain limited. The development of clinically viable predictive tools faces technical challenges requiring interdisciplinary collaboration with clinical informaticists. Clinicians’ reliance on their experience may limit trust in predictive tools, emphasizing the need for integration with clinical guidelines. Our plan for tools will be designed to support, not replace clinical judgment, enhancing decision-making through careful interpretation of outputs. We hope that in the future, there will be an emergence of more flexible and comprehensible explainability tools, or improvements in the self-explanatory ML algorithms. There is no doubt that utilizing the first ICU admission data presents limitations, as the progression of sepsis is a dynamic process. We will also investigate the influence of the dynamic trajectory of biomarkers on the prognosis of sepsis, so as to better conform to the dynamic changes of the pathophysiology of sepsis. It is worth noting that ensemble models by leveraging the strengths of multiple models, may achieve superior performance compared to individual model. We will further explore the use of ensemble techniques to optimize clinical prediction models in our future work.

## Conclusion

A unique approach was provided to simultaneously and timely distinguish multiple prognoses in sepsis. CatBoost model affords a valuable reference for the clinical evaluation and proactive intervention.

## Electronic supplementary material

Below is the link to the electronic supplementary material.

Supplementary Material 1

## Data Availability

The data generated and analyzed during the current study are available on the MIMIC-IV website at http://mimic.physionet.org/, 10.13026/6mm1-ek67. Raw data extracted in our initial stage is provided in supplementary information files.

## Abbreviations

AUC: The area under the receiver-operating-characteristic curve
SHAP: The Shapley Additive explanations
ICU: Intensive care unit
CCI: Chronic Critical Illness
SOFA: Sequential Organ Failure Assessment
qSOFA: Quick SOFA
SIRS: Systemic Inflammatory Response Syndrome
APACHE II: Acute Physiology and Chronic Health Evaluation II
ML: Machine learning
