# Supplementary material for: Harness machine learning for multiple prognoses prediction in sepsis patients: evidence from the MIMIC-IV database
Source: BMC Med Inform Decis Mak. 2025 Mar 31;25:152. doi: 10.1186/s12911-025-02976-y (PMC11959728; doi:10.1186/s12911-025-02976-y)

Additional file 1

Supplementary [Figure1](12911_2025_2976_Article.docx#LinkManagerBM_FIG_deAGDGE3).ROC curves for each prognosis of Catboost and RF in training set.


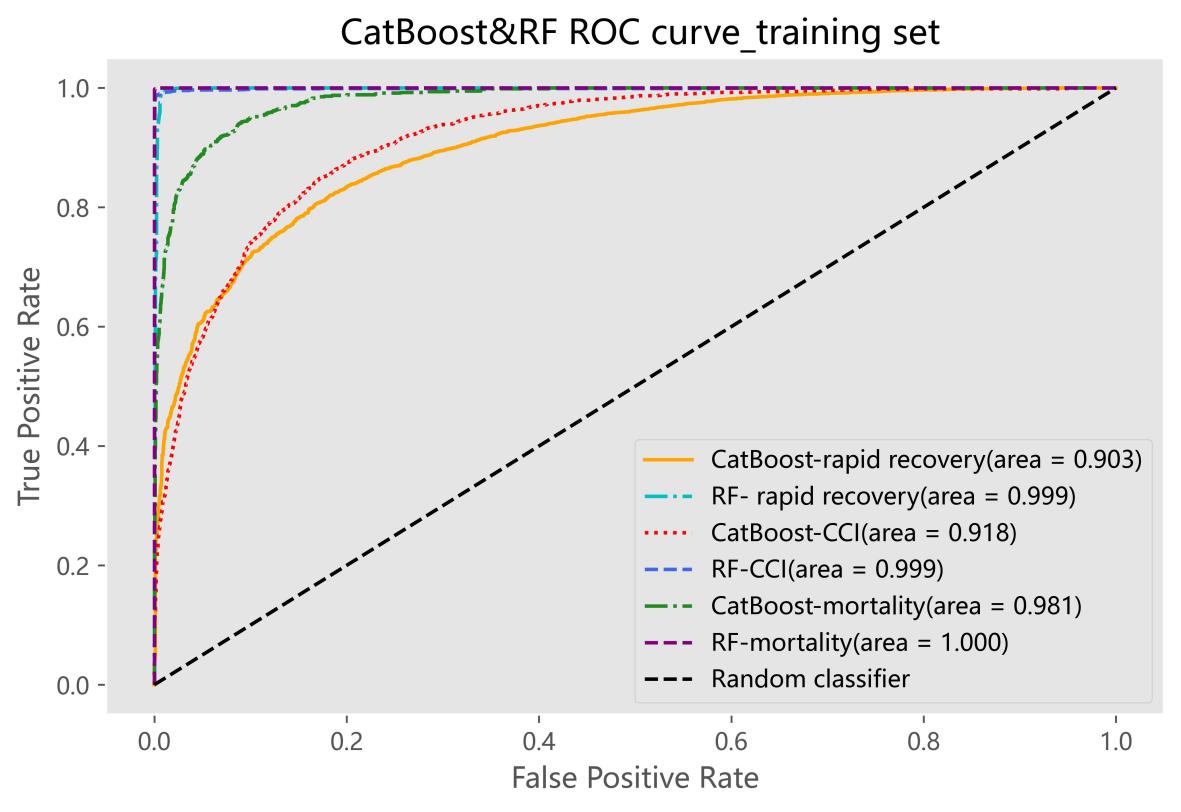


Supplementary [Figure2](12911_2025_2976_Article.docx#LinkManagerBM_FIG_5EWnrIqO).ROC and PR Curves of SOFA、APACHE II、OASIS、SIRS

、SAPSII Scores

| 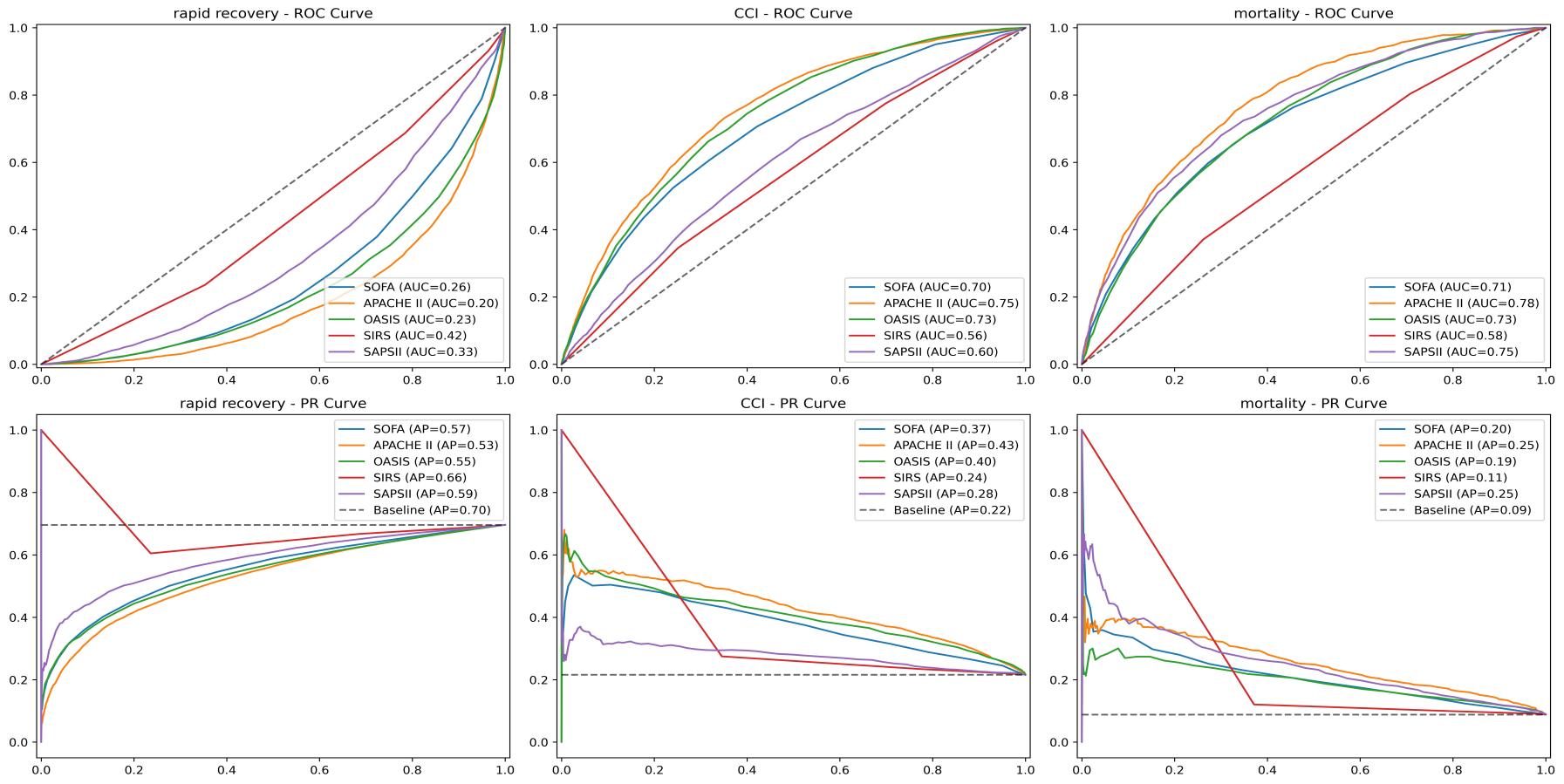 |
| --- |

Supplementary [Table 1](12911_2025_2976_Article.docx#LinkManagerBM_TABLE_fr1aqfco).Performance Metrics of Overall CatBoost Before and After Calibration with Isotonic Regression

| Calibration | Precision(95%CI) | Accuracy(95%CI) | Recall(95%CI) | F1-score(95%CI) | AUC(95%CI) |
| --- | --- | --- | --- | --- | --- |
| Before | 0.684(0.669,0.701) | 0.652(0.637,0.666) | 0.652(0.637,0.666) | 0.665(0.650,0.679) | 0.771(0.758,0.785) |
| After | 0.669(0.653,0.685) | O,696(0.682,0.710) | 0.696(0.682,0.710) | 0.677(0.662,0.693) | 0.767(0.753,0.780) |

Supplementary [Figure3](12911_2025_2976_Article.docx#LinkManagerBM_FIG_vANAyc2H).Calibration Curves for Overall CatBoost Model Using Isotonic Regression


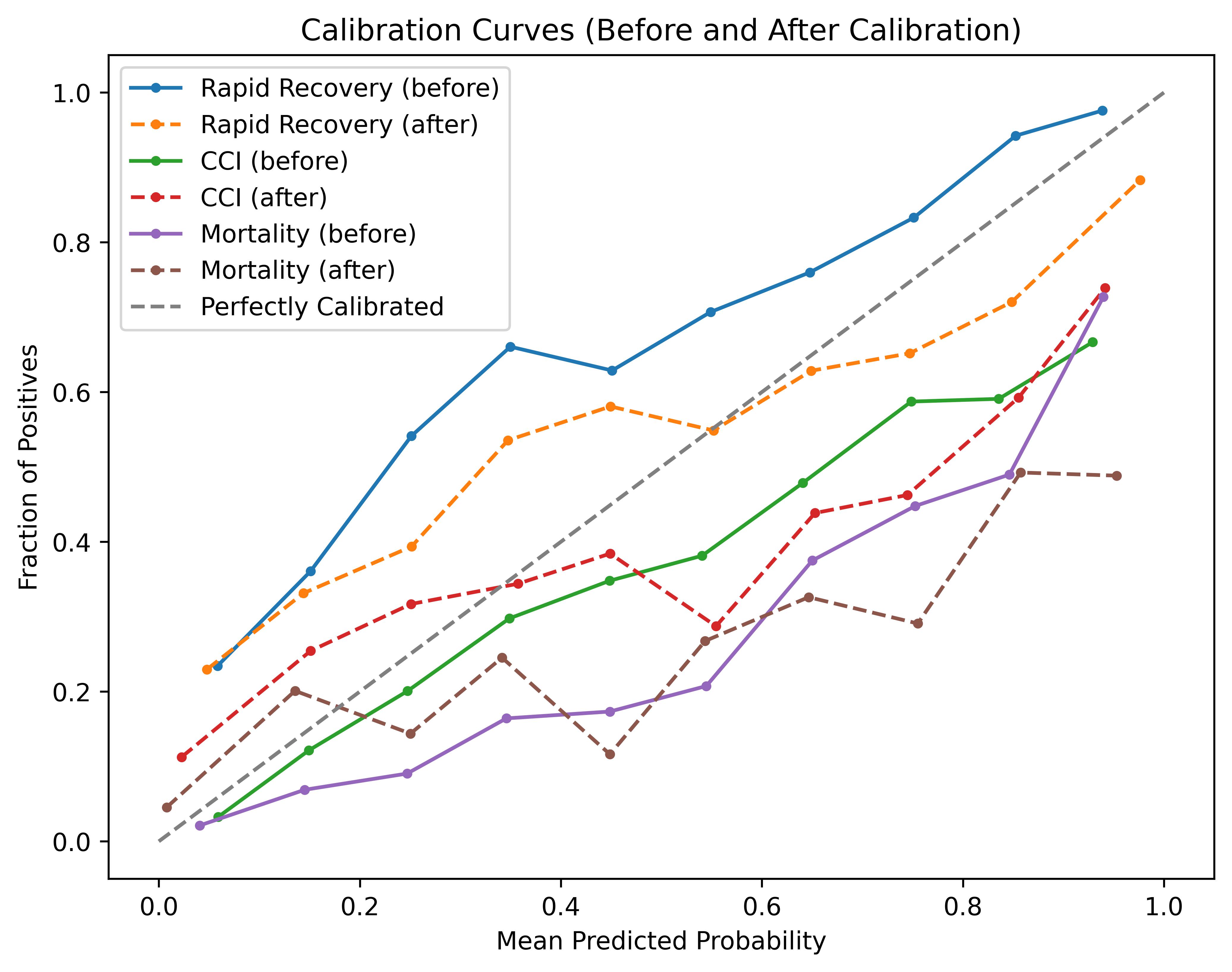


Supplementary [Figure4](12911_2025_2976_Article.docx#LinkManagerBM_FIG_dW4sB2s6).Calibration Curves for Mortality Prognosis Using Isotonic Regression


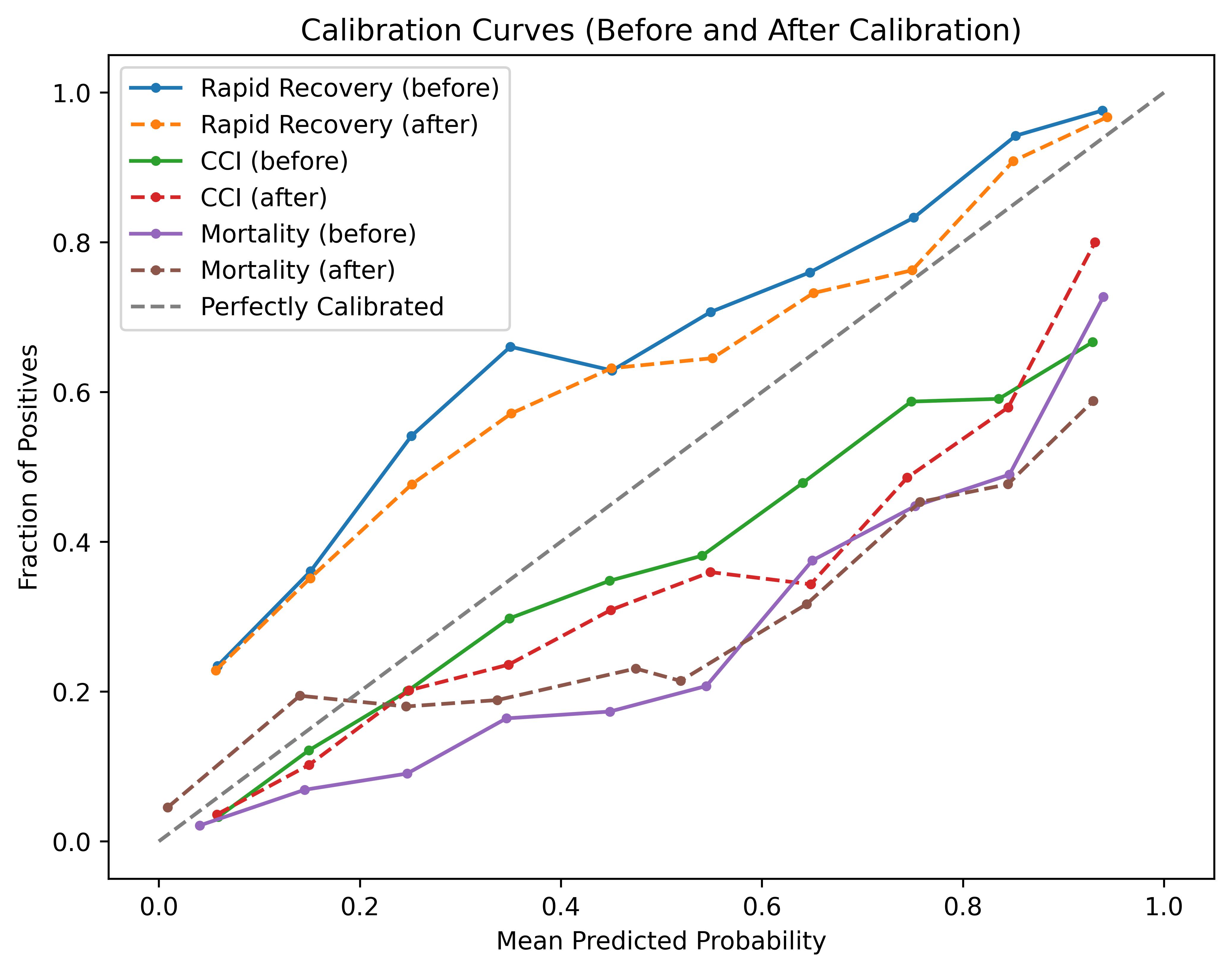

Supplement: Supplementary file 1 — Supplementary Material 1 [file 12911_2025_2976_MOESM1_ESM.docx]
